# Neural stem cells in adult neurogenesis and their therapeutic applications in neurodegenerative disorders: a concise review

**DOI:** 10.3389/fmmed.2025.1569717

**Published:** 2025-06-19

**Authors:** Teketay Bayleyegn Derso, Bemrew Admassu Mengistu, Yitayew Demessie, Melkie Dagnaw Fenta, Kalkidan Getnet

**Affiliations:** ^1^ School of Veterinary Medicine, Mersa Agriculture College, Woldia University, Wollo, Ethiopia; ^2^ Department of Biomedical Sciences, College of Veterinary Medicine and Animal Sciences, Univeristy of Gondar, Gondar, Ethiopia; ^3^ Department of Veterinary Clinical Medicine, College of Veterinary Medicine and Animal Sciences, University of Gondar, Gondar, Ethiopia; ^4^ Department of Veterinary Epidemiology and Public Health, College of Veterinary Medicine and Animal Sciences, University of Gondar, Gondar, Ethiopia

**Keywords:** adult neurogenesis, neurodegenerative diseases, neural stem cell therapy, stem cells, neural stem cells therapeutic applications

## Abstract

The idea of ​​using stem cell therapy to treat neurodegenerative diseases has undergone significant change over the years and has made significant progress recently. Neurotrophins, growth factors, and transcription factors regulate neural stem cell proliferation and differentiation. Disruption of these regulatory mechanisms, including negative feedback, can contribute to neurodegenerative diseases. Contemporary research highlights a growing global concern regarding diverse neurodegenerative disorders affecting both humans and animals. These conditions arise from neuronal cell death, axonal regeneration failure, and impairment of neuronal structure. Current pharmacological treatments primarily offer symptomatic relief without altering disease progression. Consequently, researchers are investigating innovative therapeutic strategies, with neural stem cell therapy emerging as a promising avenue. Adult neural stem cells, embryonic neural stem cells, and induced pluripotent stem cells represent potential cell sources, although challenges such as ethical considerations and technical limitations remain. The therapeutic application of neural stem cells holds significant promise for addressing neurodegenerative diseases, including Alzheimer’s disease, stroke, amyotrophic lateral sclerosis, spinal cord injury, and multiple sclerosis. Neural stem cell therapy aims to replenish lost neurons and promote neural regeneration in these conditions. While clinical trials have demonstrated some success in improving cognitive and motor functions in individuals with neurodegenerative impairments, challenges such as immunological rejection, the identification of compatible cell sources, ethical concerns, treatment efficacy, and potential side effects necessitate thorough investigation before widespread clinical implementation. Despite these challenges, neural stem cell-based therapy offers substantial potential for revolutionizing the treatment of neurodegenerative diseases and central nervous system injuries. This paper, therefore, explores adult neurogenesis and the therapeutic potential of neural stem cells within the dynamic field of neurodegenerative disorders.

## Introduction

In recent times, there has been a growing global concern regarding brain diseases affecting both humans and animals. These include neurodegenerative diseases, autoimmune disorders, brain injuries, and idiopathic conditions. The significant impact of these diseases has affected a considerable portion of the population (Ibrahim et al., 2023; Korczyn et al., 2011). Damage to the central nervous system (CNS) results from various conditions such as cell death, failure of axonal regeneration, demyelination, and overall impairment of neuronal structure and function. These conditions, whether occurring individually or in combination, whether stemming from genetic or acquired causes, and whether their origins are known or unknown, are collectively referred to as neurodegenerative disorders (Hussain et al., 2018). Neurodegenerative diseases are debilitating disorders that affect millions of people worldwide (Gholamzad et al., 2023).

To address these challenges, the researcher is increasingly exploring innovative and more efficient approaches for treating patients with neurological disorders. Therapeutic alternatives for human and animal brain diseases are actively being explored (Isaković et al., 2023). The present approved pharmacological interventions only provide relief for accompanying symptoms, and therapeutics that can alter the course of the disease are conspicuously lacking (Adugna et al., 2022; Bavarsad et al., 2023). Due to this, scholars are investigating various treatment options and interventions to address these complex challenges and enhance the wellbeing of individuals and animals affected by such conditions. Among the array of therapeutic strategies, neural stem cell therapy stands out as a highly promising option for addressing brain diseases (Wei et al., 2023; Gonzalez et al., 2014). The commonly used neural stem cell therapy for brain disease includes adult neural stem cells (NSC), embryonic neural stem cells, and induced pluripotent stem cells (Zhao et al., 2021; Latchney and Eisch, 2012; Obernier and Alvarez-Buylla, 2019). Stem cells are remarkable, undifferentiated cells found in all multicellular organisms that possess the unique ability to both divide through mitosis and differentiate into diverse specialized cell types (Avasthi et al., 2008; Bavarsad et al., 2023). According to where they originated, stem cells can be broadly classified into two groups: those from embryos and those from adults (Barky et al., 2017). Furthermore, stem cells can be divided by the extent to which they can differentiate into different cell types. These four main category are totipotent, pluripotent, multipotent, and unipotent (Kalra and Tomar, 2014).

The utilization of embryological neural stem cells for research and therapy is constrained by factors such as limited availability, strict ethical and political issues, high costs, and immune rejection. Nonetheless, scientists are actively exploring alternative approaches to address these challenges. The incorporation of these emerging methodologies within the research community will shape the future application of embryonic neural stem cells in cell-based therapeutics (Taupin, 2008; Latchney and Eisch, 2012). Various studies have demonstrated that induced pluripotent stem cells enhance both motor and cognitive function in mouse brain tissue, with these cells migrating to injured areas from the injection site. However, there is a limited amount of research on induced pluripotent stem cell therapy for brain injuries due to challenges in acquiring these cells, high therapy expenses, and technical constraints (Adugna et al., 2022).

Due to their ability to generate various cell types within the central nervous system, adult neural stem cells are an ideal tool for treating nervous system disorders. NSCs present advantages over other cell types in cell-based therapies. The discovery of neurogenesis in the adult brain and the presence of NSCs in the adult CNS offer promising prospects for cell-based therapy, with the added benefit of avoiding ethical and political concerns associated with the generation and use of these cells (Chang et al., 2024; Latchney and Eisch, 2012). Such therapeutic approaches could encompass stimulating differentiation *in vivo* and transplanting neural progenitor and stem cells sourced from the adult CNS (Taupin, 2008).

Adult neural stem cells are undifferentiated cells that have the potential to develop into various specialized cell types. They are present throughout the body following embryonic development and divide to replace dying cells and repair damaged tissues. The primary functions of adult neural stem cells in a living organism are to maintain regeneration and repair the tissue in which they reside (Jagiri et al., 2019; Latchney and Eisch, 2012; Obernier and Alvarez-Buylla, 2019). In most regions of the adult mammalian brain, neural stem cells give rise to neurons (Basak and Taylor, 2009; Terskikh et al., 2006). Neurogenic stem cells are located in the subventricular zones of the lateral ventricle, the hippocampal dentate gyrus, and other brain regions including the cerebral cortex, amygdala, hypothalamus, and substantia nigra. These cells can be isolated, cultured, and differentiated into multiple neural lineages, making them valuable for cellular-replacement therapy in treating neurological disorders (Adugna et al., 2022).

Neural stem cells represent a unique type of somatic cell that has the capacity for long-term self-renewal and the ability to generate different neural lineages (Duan et al., 2008). Vertebrate neurogenesis occurs not only during embryogenesis but also at the adult stage (Grandel et al., 2006). In rodents and songbirds, adult neurogenesis is observed in highly restricted spatial domains that produce new cells destined for distinct telencephalic regions (Hack et al., 2005; Taupin, 2009). Neuronal stem cells and progenitor cells in the ventricular and sub-ventricular zones give rise to the primary cell types of the mammalian central nervous system (Mendez-Gomez et al., 2011; Terskikh et al., 2006). Adult somatic neural stem cells contribute to the plasticity of neuronal circuits by continuously introducing immature, new neurons with distinct characteristics, such as hyper-excitability, and by forming new synaptic connections with mature neurons, in addition to their homeostatic role in the functional restoration of the brain following injury (Ming and Song, 2011).

From the 1960s, the brain stood as a different scenario in scientific understanding. Conventionally, neurogenesis was thought to occur exclusively during the embryonic development of the mammalian CNS. In the early 20th century, the prominent histologist Ramón Cajal famously asserted, “Once development ended, the sources of neuron, axonal, and dendritic growth and regeneration ceased irreversibly. In adult centers, nerve pathways were considered fixed, concluded, and unchangeable.” However, this conventional dogma was overturned in the 1960s with Joseph Altman’s ideas. Altman suggested that the incorporation of newly formed neurons could indeed take place in various areas of the adult mammalian brain, such as the hippocampus, olfactory bulb, and other brain regions (Altman and Das, 1965). In addition to this, current pharmacological interventions for brain disease offer limited relief. However, in recent years, neural stem cell therapy has emerged as a promising option due to its potential to address the underlying pathology of brain diseases (Gonzalez et al., 2014; Yuzwa et al., 2021; Gholamzad et al., 2023; Harkins et al., 2025). Therefore, the objectives of this paper were to give an overview of adult neurogenesis and highlight the promising therapeutic applications of neural stem cells for many neurological abnormalities.

## Historical background of adult neurogenesis

The field of neuroscience held the view that, in contrast to what occurs during embryogenesis and in the majority of other adult tissues, the ability of mammals’ CNS to produce new nerve cells (neurogenesis) was irreversibly stopped after birth until the early 1990s (Colucci-D’Amato et al., 2006). However, in the 1960s, Joseph Altman and coworkers found that cells in the hippocampus’s dentate gyrus could absorb radioactive thymidine, which ultimately resulted in the discovery of neurogenesis in the adult rat brain (Altman and Das, 1965). In the 1980s, Fernando Nottebohm published the first clear evidence of adult neurogenesis in songbirds. This research has completely altered a neuroscientific paradigm. It was suggested that adult songbirds’ capacity to pick up new songs demonstrated the development of new neurons in their brains, which assisted the birds in creating memories of the new song (Nottebohm, 2002). According to Liao et al. (2019) research, the human hippocampal cell can continue generating new neurons for every moment of its lifetime. It is now generally accepted that adult neurogenesis is present in the dentate gyrus and sub ventricular zone (Gonçalves et al., 2016) and various other brain regions such as the cerebral cortex, amygdala, hypothalamus, and substantia nigra of humans, most mammals, and as well various vertebrate species (Ming and Song, 2005; Taupin, 2009). The process of adult neurogenesis in fish, amphibians, reptiles, and avian brains is well characterized (Schmidt and Derby, 2011).

Adult neurogenesis refers to the formation of fully developed and functional neurons from neural stem cells within the brain. This process encompasses all stages from the initial division of a precursor cell to the establishment and viability of a mature, functioning new neuron (Abdissa et al., 2020). In the adult brain, neurogenesis is predominantly found in the dentate gyrus (DG) of the hippocampus and the subventricular zone in a variety of species, including humans (Taupin, 2006; Carlén et al., 2002). Adult neurogenesis is a multistage process that includes the proliferation, migration, survival, differentiation, and integration of newborn neurons in an already-existing system (Migaud et al., 2016; Li et al., 2018; Taupin, 2009). It is necessary to make modifications to its rates and pathways to produce the right amount of neurons to sustain brain functions due to dramatic changes in brain size and environmental specializations among various species during mammalian evolution (Loseva et al., 2009). Within the mammalian central nervous system, adult neural stem cells are unique somatic cell types capable of producing many neural lineages and engaging in long-term self-renewal (Duan et al., 2008; Terskikh et al., 2006). To date, the field of adult neurogenesis has evolved from initial skepticism to becoming a dynamic and expanding area of neuroscience. Ongoing research continues to explore the complex mechanisms, functional implications, and therapeutic potential of generating new neurons in the adult brain (Taupin, 2007; Taupin, 2009; Boldrini et al., 2018; Moreno-Jiménez et al., 2019; Harkins et al., 2025; Yuzwa et al., 2021); the detail of the summary is indicated in Table 1.

**TABLE 1:** Lists of the significant turning points and advancements in the history of adult neurogenesis: The key findings and developments that have influenced the study of adult neurogenesis during the previous few decades are listed in this table. It draws attention to the chronology of pioneering research, technical developments, and theoretical advances that have shaped our present knowledge of neurogenesis in the adult brain. Every item documents a significant turning point, outlining the experimental accomplishment as well as its wider implications for brain research. This table offers a thorough framework for understanding the evolution of knowledge in this quickly developing field by following these significant changes, from early morphological findings to contemporary molecular and cellular characterizations.

Milestone	Description	References/s
Discovery of Adult Neurogenesis	Altman and Das provided the first experimental evidence of new neuron formation in adult rats, challenging long-held beliefs that neurogenesis ceases after development	Altman and Das (1965)
BrdU as a Marker for Neurogenesis	Bromodeoxyuridine (BrdU) labeling was introduced to trace proliferating cells, enabling detailed studies of neurogenesis	Miller and Nowakowski (1988)
Environmental Enrichment Promotes Neurogenesis	Demonstrated that enriched environments significantly increase the rate of neurogenesis in the adult hippocampus	Kempermann et al. (1997)
Adult Neurogenesis in the Human Hippocampus	Confirmed for the first time that neurogenesis occurs in the adult human hippocampus, opening new avenues for brain repair research	Eriksson et al. (1998)
Mechanisms of Neurogenesis in Songbirds	Provided mechanistic insights into neurogenesis in the adult brain using songbirds, a pivotal model for studying neuronal plasticity	Nottebohm (2002)
GABAergic Input to New Neurons	Found that newly generated neurons in the adult hippocampus receive excitatory GABAergic inputs essential for maturation and integration	Tozuka et al. (2005)
Isolation of Neural Stem Cells	Described methods to isolate and characterize adult neural progenitor and stem cells from the rodent hippocampus	Taupin, (2006); Taupin, (2009)
Adult Neurogenesis in the Human SVZ	Identified and characterized adult neural stem cell niches in the human subventricular zone (SVZ)	Curtis et al., (2007a); Bonafina et al., (2020)
Single-cell RNA-seq of Neural Stem Cells	Revealed heterogeneity among hippocampal neural stem cells using single-cell transcriptomics, refining understanding of stem cell dynamics	Hochgerner et al. (2018)
Persistence of Neurogenesis in Aging	Studies reignited debate by showing neurogenesis persists in aged human brains, though rates decline with age	Boldrini et al. (2018); Moreno-Jiménez et al. (2019)
Spatial Transcriptomics in Neurogenesis	Advanced spatially resolved transcriptomic mapping of neurogenic niches, offering insights into microenvironmental influences on stem cells	Harkins et al. (2025); Picard‐Riera et al. (2004)
Immune System’s Role in Neurogenesis	Recent studies highlighted the modulation of adult neurogenesis by immune cells, cytokines, and inflammatory processes	Teperikidis et al. (2023); Greenhalgh et al., (2023)

## Common locations of adult neurogenesis

### Adult neurogenesis in the olfactory bulb

The olfactory lobe emerges from the rostral-ventral portion of the telencephalic vesicle and can be divided into two principal parts: a posterior part including the lobe of the hippocampus, and an anterior part including the olfactory bulb, tubercle, and peduncle (Pagano et al., 2000; Carlén et al., 2002; Curtis M. A. et al., 2007). The olfactory bulb is a vital structure in the vertebrate brain responsible for processing and interpreting olfactory information, or the sense of smell. Situated in the forebrain, the olfactory bulb receives input from the olfactory receptors in the nasal cavity and plays a crucial role in the initial stages of odor perception (Gheusi and Lledo, 2014).

The process of generating new neurons occurs in the olfactory bulb throughout an individual’s life, a phenomenon known as adult neurogenesis. This unique characteristic sets the olfactory bulb apart from many other brain regions that generally exhibit limited neurogenesis in adulthood. The continuous generation of new neurons in the olfactory bulb is essential for the maintenance and adaptation of the olfactory system (Okano and Sawamoto, 2008). In the adult mammalian brain, neurogenesis in the olfactory bulb primarily involves the generation of new neurons from neural stem cells located in the subventricular zone. These newly formed neurons migrate to the olfactory bulb, integrate into existing neural circuits, and contribute to the modulation of olfactory function. This ongoing neurogenesis is believed to be involved in the plasticity of olfactory circuits, allowing the brain to adapt to changing olfactory environments and experience (Picard‐Riera et al., 2004; Bonafina et al., 2020). Adult-born olfactory bulb interneurons largely derive from the adult neural stem cells in the subventricular zone and continually integrate into the neuronal circuitry within the olfactory bulb, facilitating olfactory information processing and maintaining the functional plasticity of the olfactory bulb (Paß et al., 2020).

Knowledge about the mechanisms and significance of neurogenesis in the olfactory bulb is a fascinating area of research, shedding light on the dynamic nature of the adult brain and its ability to continually generate new neurons to maintain sensory function. This process holds an implication not only for our understanding of olfaction but also for broader neuroscientific inquiries related to neural plasticity, learning, and memory in the adult brain (Taupin, 2007; Taupin, 2009).

The subventricular zone (SVZ) of the olfactory bulb is a dynamic and crucial region within the vertebrate brain that plays a central role in the ongoing generation of neurons in the adult mammalian brain. SVZ is a paired brain structure situated throughout the lateral walls of the lateral ventricles. It contains the largest population of proliferating cells and is populated by heterogeneous populations of stem and progenitor cells (Alvarez-Buylla and Garcıa-Verdugo, 2002; Abdissa et al., 2020). The lateral ventricles are lined with an abundance of neural stem cells referred to as type-B1 cells, which resemble astrocytes and differentiate into neurons that populate the olfactory bulb. Glial fibrillary acidic protein (GFAP), glutamate aspartate transporter, and brain lipid-binding protein are expressed by type-B1 cells. Activated type-B1 cells express nestin and divide asymmetrically for self-renewal or to give rise to achaetescute homolog one and distal-less homeobox two-expressing C cells (Jurkowski et al., 2020). SVZ neurogenesis consists of sequential cell functions, including NSC activation, transit-amplifying progenitor cells proliferation, neuroblast migration, and differentiation. The NSCs exhibit apical-basal polarity with an apical primary cilium contacting the cerebrospinal fluid and a basal process contacting a blood vessel (Sun et al., 2022).

**FIGURE 1:**
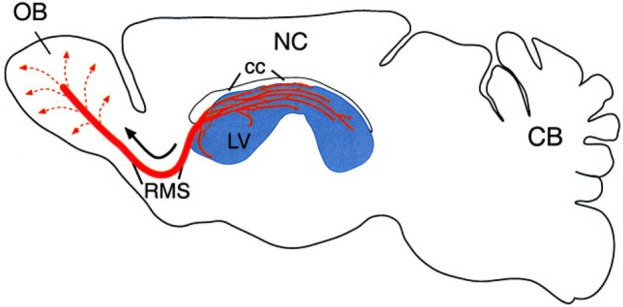
This sagittal view of the adult rodent brain (olfactory bulb on the left, cerebellum on the right) illustrates the subventricular zone-olfactory bulb (SVZ-OB) system. The SVZ, located along the lateral ventricle (LV, shown in blue), is a site of continuous neurogenesis. Newly generated neurons (A cells) organize into migrating chains (red lines) that form an intricate network within the SVZ. A significant portion of these chains in the anterior SVZ connect to the rostral migratory stream (RMS), which serves as a pathway for young neurons to reach the olfactory bulb’s core. Once there, these cells disperse radially (dotted lines) and mature into granule and periglomerular interneurons. The diagram also shows the neocortex (NC) and corpus callosum (cc) (Alvarez-Buylla and Garcıa-Verdugo, 2002; Curtis et al., 2012).

**FIGURE 2:**
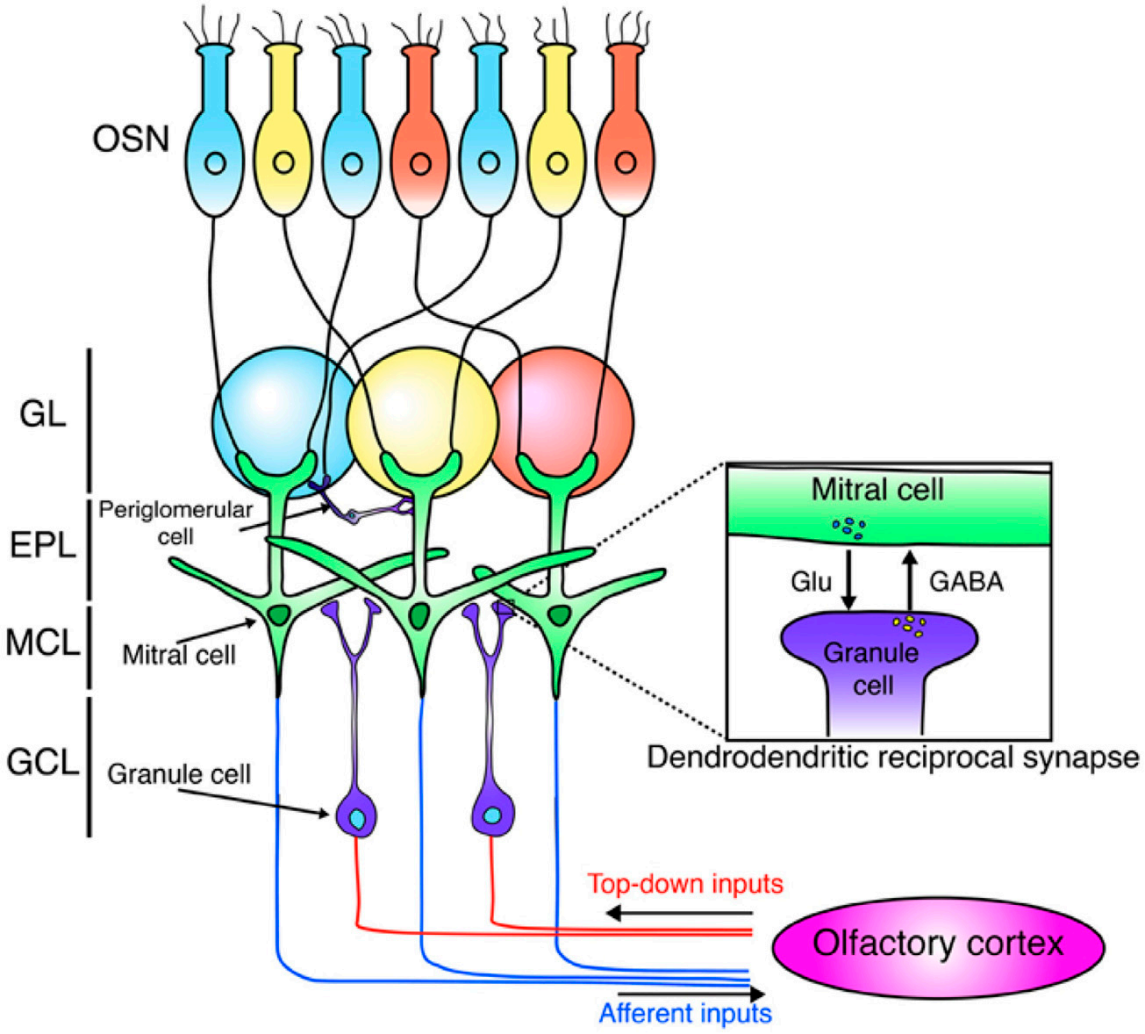
It is a schematic depicting the olfactory bulb’s neural network. Olfactory sensory neurons (OSNs), tuned to the same odors (indicated by blue, yellow, and red), project to and synapse within the same glomeruli. These OSNs excite mitral cells, the primary output neurons that project to the olfactory cortex. Mitral cells also interact locally with granule cells via dendrodendritic synapses. Granule cells receive excitatory input from the olfactory cortex. The diagram identifies olfactory sensory neurons (OSN) and the olfactory bulb layers: glomerular layer (GL), external plexiform layer (EPL), mitral cell layer (MCL), and granule cell layer (GCL) (Sakamoto et al., 2014).

In the olfactory system, cells originate in the SVZ lining the lateral ventricles and then undergo migration up to 5 mm (in mice) along a clearly defined pathway known as the rostral migratory stream (Figures 1, 2). This journey leads them into the olfactory bulb, where they undergo differentiation into either granule cells or granule progenitor cells (Lledo et al., 2006). Notably, approximately 95% of the newly formed neurons in the olfactory bulb develop into granule cells (Lledo and Saghatelyan, 2005). The minority that differentiates into granule progenitor cells, however, gives rise to all identified molecular and morphological subtypes of progenitor cells (Bagley et al., 2007; Batista-Brito et al., 2008). During the early postnatal period, additional migratory pathways emerge from the SVZ of GABAergic cells into various forebrain regions, including the cortex, striatum, and nucleus accumbens (Inta et al., 2008).

While the functional importance of subventricular zone neurogenesis has been comparatively less studied than that of hippocampal neurogenesis, it is noteworthy that SVZ neurogenesis persists throughout adulthood in the mammalian brain. This ongoing process significantly contributes to the establishment and refinement of the optimal olfactory circuitry. Through constant granule cell regeneration and replacement, mammals can respond to new environmental stimuli and reinforce particular odorant representations that are more pervasive in their environment (Jurkowski et al., 2020; Bonafina et al., 2020; Bragado Alonso et al., 2019). Neuroblasts originating from the subventricular zone exhibit migration not only towards the olfactory bulb (OB) but also extend beyond the rostral migratory stream (RMS) to reach the basal ganglia and cerebral cortex (Curtis M. A. et al., 2007). This holds importance as movement disorders like Parkinson’s disease involve degeneration in parts of the basal ganglia, and alzheimer’s disease sees specific cortical regions degenerating. The potential for neural stem cells to enter these affected areas through the RMS opens up possibilities for the development of novel treatments for neurodegenerative diseases (Abdissa et al., 2020; Korczyn et al., 2011). The ongoing generation of interneurons in the olfactory bulb via subventricular zone neurogenesis plays an essential role in maintaining olfactory function. These interneurons establish synapses with olfactory bulb projection neurons, which, in turn, receive signals from olfactory sensory neurons located in the olfactory epithelium (Chen et al., 2023; Alvarez-Buylla and Garcıa-Verdugo, 2002; Sakamoto et al., 2014; Curtis M. A. et al., 2007), depicted in the following Figures 1, 2.

### Adult neurogenesis in the hippocampus

According to the conventional perspective, neurogenesis, or the formation of new neurons, occurred exclusively during embryonic and early developmental stages, with little to no regeneration in the adult brain (Figure 2). However, groundbreaking research over the past few decades has challenged this notion, revealing that certain regions of the adult mammalian brain, particularly the hippocampus, retain the ability to produce new neurons throughout life. Nowadays, most researchers confirm that adult neurogenesis takes place in the dentate gyrus (DG) of most mammals, including humans (Gonçalves et al., 2016; Boldrini et al., 2018; Hochgerner et al., 2018; Moreno-Jiménez et al., 2019). The discovery of adult hippocampal neurogenesis has sparked a paradigm shift in our understanding of brain plasticity and has profound implications for cognitive and emotional functions. The hippocampus is a key player in various cognitive processes, including spatial navigation, pattern recognition, and the formation of declarative memories. The integration of newly generated neurons into existing neural circuits is thought to contribute to these cognitive functions, providing a dynamic and adaptive mechanism for the brain to respond to environmental stimuli and experiences (Kozareva et al., 2019).

Sub-granular zone of the hippocampus is found deep within the hippocampal parenchyma at the interface between the hilus and the granular cell layer of the DG of the hippocampus. It contains a population of neuronal precursor cells in the DG that generate large numbers of new granule neurons throughout adulthood (Abdissa et al., 2020). The dentate gyrus constitutes a V-shaped formation within the hippocampus, situated in the medial temporal cortex of mammals. Adult neurogenesis in the dentate gyrus begins with a precursor population located in the subgranular zone (SGZ), a narrow tissue band between the granule cell layer and the hilus. The majority of cell proliferation takes place in this region (Ehninger and Kempermann, 2008).

Mammalian adult hippocampal neurogenesis refers to the process by which new neurons are generated in the hippocampus, a crucial region of the brain involved in learning, memory, and emotional regulation (Kronenberg et al., 2003). The process of adult hippocampal neurogenesis involves several distinct stages, including the proliferation of neural stem cells, their differentiation into immature neurons, migration to specific regions within the hippocampus, and finally, their integration into existing neural networks. Numerous factors, both intrinsic and extrinsic, regulate these stages (Picard‐Riera et al., 2004). The process of adult hippocampal neurogenesis involves multiple continuous developmental stages in the adult brain that lead to the formation of new neurons. These stages include the precursor stage, early survival stage, post-mitotic maturation stage, and late survival stage (Kozareva et al., 2019; Encinas and Fitzsimons, 2017). The precursor cells of the dentate gyrus are located in the SGZ, situated between granule cells and the hilus. SGZ contains radial glia-like stem cells called type-1 cells, which share astrocytic properties, express GFAP, and have a radial morphology. Type-1 cells infrequently divide and may be quiescent (Kempermann, 2022), as illustrated in Figure 2.

Radial glial-like cells generate intermediate progenitor cells during Stage two (differentiation), exhibiting transient amplifying traits, actively dividing while expressing either doublecortin or polysialylated neural cell adhesion molecule. These intermediate progenitor cells have the potential to transition into Stage three, marked by the migration of neuronal lineage committed cells or neuroblasts, possibly indicating the expression of cell adhesion molecules (Kumar et al., 2019; Li et al., 2014). As they progress to Stage four, mature dentate gyrus neurons undergo axonal and dendritic targeting, expressing calcium-binding proteins and neuron-specific nuclear proteins, serving as post-mitotic neuronal markers. Upon reaching maturity, these newly formed granule cells seamlessly integrate into the hippocampal circuitry during Stage five, characterized by synaptic integration. These integrated neurons become actively involved in influencing hippocampal functions such as learning, memory, and spatiomotor performance (Von Bohlen Und Halbach, 2007).

The fundamental concepts of maturation and synaptic integration in the dentate granule of the hippocampal formation, the other site of ongoing adult neurogenesis in mammals, are comparable to those previously discussed for the olfactory system, but there are several significant differences (Whitman and Greer, 2009; Curtis et al., 2012). The hippocampus is a crucial region for these processes. On the other hand, olfactory bulb neurogenesis is more directly related to olfactory function and the processing of olfactory information. Newly generated neurons in the adult hippocampus typically migrate a short distance and integrate into existing hippocampal circuits. In contrast, newly generated neurons in the subventricular zone migrate a much longer distance along the rostral migratory stream to reach the olfactory bulb, where they differentiate into interneurons (Zhao et al., 2006). Granule cells in the dentate gyrus are the sole type of neuron produced by adult hippocampal neurogenesis (Rietze et al., 2000; Liu et al., 2003).

The rate of neurogenesis differs between the two regions. Adult hippocampal neurogenesis is relatively modest and declines with age, while olfactory bulb neurogenesis persists throughout adulthood. In contrast to the subgranule zone, which produces excitatory dentate gyrus granule neurons, adult neurogenesis in the SVZ mostly produces inhibitory olfactory bulb interneurons (Ghosh, 2019). Understanding the mechanisms and regulation of neurogenesis in the hippocampus is a topic of ongoing research, as it has implications for conditions such as alzheimer’s disease, depression, and other neurological disorders. The ability of the adult brain to generate new neurons in the hippocampus provides hope for potential therapeutic strategies to enhance cognitive function and treat certain neurological conditions (Zhao et al., 2021; Matsubara et al., 2021).

### Adult neurogenesis in other brain regions

It has been proposed that small amounts of new neurons are produced in other regions of the brain in addition to the neurogenesis in the subventricular zone and dentate gyrus. These regions comprise the brain’s neocortex, striatum, amygdala, substantia nigra, third and fourth ventricles. The development of neurospheres in these areas indicated the presence of neural stem cells (Ming and Song, 2005; Curtis et al., 2012).

### Molecular biomarkers to detect cell types in neurogenesis

**FIGURE 3:**
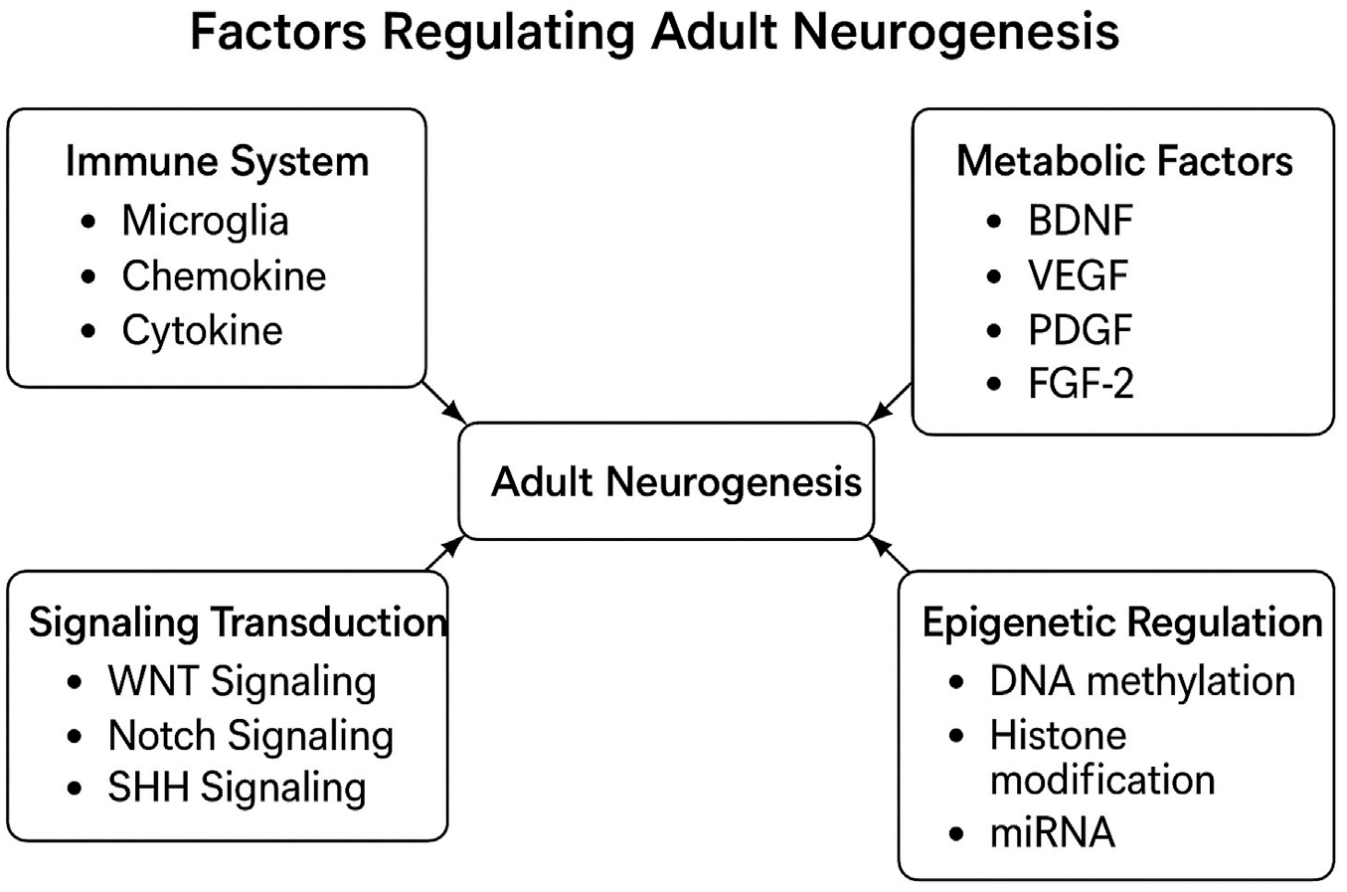
This diagram illustrates factors regulating adult neurogenesis. It categorizes and summarizes key influences like immune responses and metabolic factors. This visual reference clarifies complex interactions among these elements (Horgusluoglu et al., 2017).

In the past, the first initial confirmation of human adult neurogenesis was based on bromodeoxyuridine pulse-chase experiments. These experiments are commonly used to label dividing cells in living tissue and track their descendants by immunofluorescent labeling of fixed tissue. Using this bromodeoxyuridine method, researchers demonstrated neurogenesis in a small set of *postmortem* hippocampal samples from cancer patients who had received bromodeoxyuridine injections for diagnostic purposes (Lucassen et al., 2020). Nowadays, there are several multiple immunocytochemical markers, like Ki-67 as mitotic markers, SOX2 and brain lipid binding protein (BLBP) as markers for radial glia-like stem cells, and DCX and poly-sialylated-neural cell-adhesion molecule (PSA-NCAM) as markers for early immature neurons, and electron microscopy was employed to identify specific stages of neurogenesis (Ming and Song, 2005; Li et al., 2008; Encinas and Fitzsimons, 2017), and depicted in Table 2; Figure 3.

**TABLE 2:** The following Table summarizes key molecular biomarkers for identifying cell types involved in adult neurogenesis.

S/N	Cell type	Key molecular biomarkers
1	Neural Stem Cells	GFAP, Nestin, Prominin, SOX-2
2	Proliferating Cells	Ki-67, BrdU, PCNA
3	Immature Neurons	β-Tubulin III, DCX, PSA-NCAM
4	Radial Glial Cells	GLAST, RC2
5	Mature Neurons	NeuN, MAP-2, Neurofilament (NF), BLBP
6	Astrocytes	GFAP, S100β, GLAST, Vimentin
7	Oligodendrocytes	O4, Myelin Basic Protein (MBP), RIP

N.B. GFAP (Glial Fibrillary Acidic Protein) marks astrocytes and neural stem cells. BrdU (5-Bromo-2′-deoxyuridine) labels proliferating cells’ DNA, in neurogenesis studies. PCNA (Proliferating Cell Nuclear Antigen) indicates cell division, while DCX (Doublecortin) identifies migrating immature neurons. PSA-NCAM (Polysialylated Neural Cell Adhesion Molecule) relates to neural plasticity in immature neurons. GLAST (Glutamate Aspartate Transporter), NeuN (Neuronal Nuclei), MAP2 (Microtubule-Associated Protein 2), and NF (Neurofilament) mark glial lineage, mature neurons, dendritic development, and mature neurons, respectively. BLBP (Brain Lipid-Binding Protein), O4 (Oligodendrocyte Marker O4), and MBP (Myelin Basic Protein) identify glial progenitors, pre-oligodendrocytes, and myelinating oligodendrocytes in the CNS (central nervous system) (Li et al., 2008; Li et al., 2014).

### Adult neurogenesis regulation

Understanding the regulation of adult neurogenesis in the hippocampus, olfactory bulb, and other brain regions is an impressive step in investigating the complexities of neural plasticity. This dynamic process is bidirectionally influenced by a complex of factors, both intrinsic and extrinsic, shaping the balance between cell proliferation, differentiation, and survival (Balu and Lucki, 2009; Encinas and Fitzsimons, 2017; Matsubara et al., 2021). A more comprehension of the role and functionality of each modulator in controlling the fate and integration of neural stem cells as they develop into neurons in the subgranular zone and olfactory bulb could offer vital insights, potentially concrete the way for innovative therapies in the treatment of neurological diseases in mammals (Hussain et al., 2024).

Intrinsic factors, rooted in genetic programming, guide the fundamental processes of neural development and differentiation in the hippocampus and olfactory bulb (Balu and Lucki, 2009). The process of neurogenesis is dynamic and tightly regulated by a multitude of external as well as internal factors. These external manipulations are known to both positively and negatively impact levels of neurogenesis throughout the life of a mammal. Some of the most prominent positive regulators of hippocampal neurogenesis include exposure to an enriched environment, voluntary exercise, and diet. In contrast, factors such as inflammation, aging, and stress have been shown to dramatically reduce levels of neurogenesis (Gould, 2007). Recent research has highlighted five crucial regulators of neurogenesis, encompassing signaling transduction pathways, the vascular and immune systems, metabolic factors, and epigenetic regulation (Figure 3). The modification in these regulators during adult neurogenesis could potentially be linked to the onset of neurodegenerative diseases (Horgusluoglu et al., 2017).

The Sonic hedgehog signaling pathway assumes a crucial function in regulating developmental neurogenesis and impacting adult neurogenesis in the SVZ. Investigations focused on altering sonic hedgehog signaling functions, either by gain or loss, have demonstrated its indispensable role in regulating adult neurogenesis within the SVZ, utilizing transgenic mouse models (Jin et al., 2003). Furthermore, sonic hedgehog signaling is implicated in both neurogenesis and neurorepair, supported by evidence that administering a smoothened agonist enhances behavioral recovery in mice following a stroke and facilitates neurogenesis (Gonzalez-Reyes et al., 2019).

Neurotrophic factors are vital extracellular signaling molecules with important roles in both the developing and adult central nervous system. There are several classes of these factors, including neurotrophins (such as nerve growth factor, brain-derived neurotrophic factor, neurotrophin-3, NT-4, and NT-5, which activate signaling pathways through tyrosine kinase. Endothelial cells secrete brain-derived neurotrophic factor to support the proliferation, differentiation, and survival of neural progenitor cells during adult neurogenesis (Hussain et al., 2024).

Wnt (wingless) signaling exerts influence on adult hippocampal neurogenesis across molecular, cellular, and behavioral dimensions. This signaling pathway governs the proliferation and fate determination of neural stem cells in the context of hippocampal neurogenesis. Furthermore, neural stem cells in both the ventricular-subventricular zone and subgranular zone exhibit the capacity for self-renewal and proliferation, responding to canonical Wnt signaling to generate and expand neural progenitor cells (Horgusluoglu et al., 2017; Curtis et al., 2012).

Notch signaling plays a crucial role in sustaining and guiding the differentiation of neural stem cells during adult neurogenesis. Comparably, notch1 is essential for the self-renewal and preservation of neural stem and progenitor cells within the dentate gyrus of the hippocampus (Ables et al., 2010). The coordinated interplay between notch signaling and EGFR signaling regulates the equilibrium of neural stem cells and progenitor cells, influencing their quantity and self-renewal dynamics in the subventricular zone region (Aguirre et al., 2010; Curtis et al., 2012).

The signaling pathway of vascular endothelial growth factor (VEGF) plays an important role in various processes, including the proliferation and survival of NSCs, as well as the migration and maturation of neural progenitors (Wittko et al., 2009). VEGF, a glycoprotein, is essential for angiogenesis and vascular formation. Within the VEGF gene, there are four isoforms: VEGF-A, VEGF-B, VEGF-C, and VEGF-D. Under normal conditions, the highest levels of mRNA were observed in the olfactory bulb and dentate gyrus, which are two regions associated with adult neurogenesis. Consequently, we posit that Vascular endothelial growth factor signaling may contribute to the generation of new neurons from neural stem cells in the adult brain (Schänzer et al., 2004), as shortly illustrated in Figure 3.

### Therapeutic application of neural stem cells

Among different organs, the brain is the most sensitive organ to various disorders such as ischemic stroke, trauma, infection/inflammation, aging, and degeneration. In the brain, neurons exhibit varying degrees of susceptibility to injuries, with differences in vulnerability observed even among neuronal populations (Abe et al., 2012). Neurodegenerative diseases lead to a gradual deterioration of brain functionality secondary to neuronal and other cell loss in the central nervous system (Gholamzad et al., 2023). Importantly, abnormal protein accumulation in the brain or tissue is a hallmark of neurodegenerative diseases. Examples include β-amyloid in alzheimer’s disease, misfolded huntington protein in Huntington’s disease, ubiquitinated protein aggregation in amyotrophic lateral sclerosis, tau and β-amyloid accumulation in multiple sclerosis plaques, α-synuclein accumulation in parkinson’s disease, and tau neurofibrillary tangles in traumatic brain injuries (Hussain et al., 2018). Among the varieties of neurodegenerative disorders, the most prevalent ones are multiple sclerosis, alzheimer’s disease, parkinson’s disease, and Huntington’s disease (Thies and Bleiler, 2012; Korczyn et al., 2011).

It has been postulated that pharmacological interventions for brain disease offer limited relief. However, a previous study showed that neural stem cell therapy emerges as a promising option and a swiftly advancing field in medicine that shows immense potential for addressing a diverse neurodegenerative diseases (Mattson, 2001; Liu and Martin, 2004; Gonzalez et al., 2014). NSCs possess the remarkable abilities of proliferating, self-renewing, and differentiating into neurons, astrocytes, and oligodendrocytes. Studies have shown that transplanted NSCs are involved in the process of tissue repair and mitigation of neurodegeneration in various CNS diseases and injuries through cell replacement and neuronutrition (Gholamzad et al., 2023). In this sense, neural stem cell research has attracted significant attention in the public’s eyes (Conti and Casarosa, 2014).

The process of using new cells or tissues to replace deteriorating or degenerated ones is called cellular therapy (Taupin, 2006). NSC treatment can either include increasing endogenous neural progenitor and stem cells or transferring adult-derived neural progenitor and stem cells to repair the disorder pathways (Arvidsson et al., 2002). The discovery that neural stem cells are present in the adult central nervous system of mammals and that neurogenesis occurs in the adult brain has profound implications for cell therapy and our understanding of developmental processes (Jin et al., 2003). The major method of cellular treatment is cell transplantation, which substitutes for repairs or enhances the activities of the diseased nervous system using either neurogenic or non-neurogenic cells (Zhao et al., 2021).

In veterinary medicine, regenerative medicine employing diverse stem cells is are emerging therapeutic approach for many more kinds of refractory diseases. Since the early 2000s, cell therapy has been used clinically in veterinary medicine; the first clinical applications of cell therapy were for the treatment of tendon injuries in horses (Markoski, 2016). Adult-derived mesenchymal stem cells have been used in veterinary stem cell therapy applications to treat animals with injuries or defects affecting bone, cartilage, ligaments, or tendons to promote tissue regeneration (Koch and Betts, 2007; Gonzalez et al., 2014). The advancement of stem-cell therapies in veterinary medicine can benefit horses, dogs, and cats. These therapies can target a variety of diseases and injuries in large animals (Avasthi et al., 2008). Ribitsch et al. (2010) noted that species-specific challenges, high therapy costs, and stem cell yield must all be considered when developing neural stem cell therapies.

### Clinical applications of neural stem cells

Neural stem cells in alzheimer’s disease: Neural stem cell therapy shows promise as a treatment for a broad spectrum of neurological disorders, among them, alzheimer’s disease is characterized by neurodegeneration, resulting in cognitive decline and memory impairment. While a definitive cure for alzheimer’s disease remains elusive, existing treatments such as medications and behavioral interventions can mitigate its progression. Neural stem cell therapy is emerging as a potential avenue to ameliorate alzheimer’s disease symptoms by aiming to replenish lost neurons responsible for cognitive deficits (Table 2). Stem cells possess the capability to differentiate into diverse cell types, including neurons and glial cells, offering potential for neural regeneration (Gholamzad et al., 2023).

Neural stem cells in stroke: Stroke is a leading cause of mortality and disability globally, with more than eighty percent of strokes becoming ischemic strokes (Jiao et al., 2021). Ischemic stroke originates from the blockage of a cerebral artery and causes endothelial cells, oligodendrocytes, astrocytes, and various neuron types to die, as well as localized tissue loss. Neuronal plasticity and reorganization of neural circuitries contribute to spontaneous recovery to varying degrees, but most patients exhibit persistent motor, sensory, or cognitive impairments (Lindvall and Kokaia, 2010). The treatment of strokes will be revolutionized by exploring new strategies to aid in repair since the central nervous system has insufficient ability for self-repair (Barker et al., 2018). In the stroke-damaged mouse brain, various neural stem cells and their derivatives from both human and rodent sources can persist, develop into neurons, and repair function following transplantation (Locatelli et al., 2009).

Neural stem cells in amyotrophic lateral sclerosis: In amyotrophic lateral sclerosis, the malfunction and degeneration of motor neurons in the spinal cord, cerebral cortex, and brainstem lead to rapidly progressing muscle weakness and eventual fatality within a few years. As a matter of fact, there is no effective pharmaceutical treatment for amyotrophic lateral sclerosis. Stem cells derived from various sources, including mouse and human embryonic stem cells (Wichterle et al., 2002), as well as neural stem cells obtained from fetal rat spinal cord and human forebrain cells (Jordan et al., 2009), have been utilized to generate motor neurons *in vitro*. Motor neuron precursors and neuroblasts derived from stem cells have demonstrated the ability to establish functional synapses with muscle fibers *in vitro* (Harper et al., 2004). Moreover, when transplanted into the spinal cord of adult rats with motor neuron injuries, these stem cell-derived cells extend axons to ventral roots (Karumbayaram et al., 2009; Curtis M. A. et al., 2007), forming neuromuscular junctions with host muscles and contributing to partial recovery from paralysis. Although there’s no cure for amyotrophic lateral sclerosis (ALS), stem cell research offers hope for new therapies and regenerative strategies to combat the disease (Deshpande et al., 2006).

Neural stem cells in spinal cord injury: One of the most dangerous neurological diseases across the world is spinal cord injury. Spinal cord injury places a significant financial burden on society because of its high rate of impairment. Although there is a lack of effective therapies for it (Gao et al., 2020). Recent advancements in neural stem cell biology have created new opportunities for therapeutic approaches that use neural stem cell transplantation to replace lost brain cells in a variety of central nervous system diseases. Neural progenitor cell transplantation offers plenty of promise for the regeneration and repair of spinal cord injury (Cummings et al., 2005; Jia et al., 2020; Li et al., 2017; Van Laar et al., 2014). Several cell types, including genetically engineered fibroblasts, olfactory ensheathing cells, and neural stem cells, have been utilized to promote axonal regeneration for spinal cord injury (Teng et al., 2002; Li et al., 2017).

Neural stem cells in multiple sclerosis: Around the world, millions of people suffer from multiple sclerosis, a long-term autoimmune disease. The myelin sheath wrapping nerve fibers is damaged by multiple sclerosis, resulting in various symptoms including muscular weakness, stiffness, and problems with sight. Currently, there is no remedy for multiple sclerosis, despite great advancements in treatment (Gholamzad et al., 2023). To date, neural stem cell therapy has emerged as a promising alternative approach for treating multiple sclerosis. By utilizing stem cells’ inherent potential for repairing and regeneration, clinically successful NSC therapy for multiple sclerosis could mark a significant advancement in the field of regenerative neurology and offer a novel therapeutic choice (Pluchino et al., 2020). The overall detail has been summarized in Table 3 with some reports on clinical trials of neural stem cell therapy for various neurodegenerative diseases.

**TABLE 3:** Key techniques for studying adult neurogenesis and their application to human research: Major findings.

Pivotal finding	Methods	Study
∼700 new neurons added daily in the hippocampus; slight age-related decline	Carbon-14 dating	Spalding et al. (2013)
Human hippocampal neurogenesis persists from age 14 to 79	Immunohistochemistry, unbiased stereology	Boldrini et al. (2018)
Sharp decline in neurogenesis during the first year of life; undetectable in adults	Immunohistochemistry	Sorrells et al. (2018)
Abundant adult hippocampal neurogenesis in healthy individuals; steep drop in Alzheimer’s disease	Immunohistochemistry, optimized tissue processing	Moreno-Jiménez et al. (2019)
Persistent hippocampal neurogenesis in aged adults and Alzheimer’s patients	Immunohistochemistry	Tobin et al. (2019)
Murine-like features of neurogenesis in the human hippocampus throughout the lifespan (0–100 years)	Immunohistochemistry	Knoth et al. (2010)
Evidence of neurogenesis in the adult human striatum	Carbon-14 dating	Ernst et al. (2014)
Major decline in neurogenesis in SVZ and SGZ during early childhood	Immunohistochemistry	Dennis et al. (2016)
Minimal but persistent hippocampal neurogenesis in adults; significant presence of newborn glia	Not specified	Kumar et al. (2019)

### Recent advances and novel insights in neural stem cell therapies

Recent advancements in the domain of neural stem cell (NSC) therapies have substantially revolutionized the paradigm of regenerative medicine by elucidating therapeutic mechanisms that transcend conventional cell replacement strategies. Initially envisioned to substitute damaged neurons, NSCs are now recognized to induce significant “bystander effects” that promote neuroprotection, modulate immune responses, and augment neuroplasticity (Smith et al., 2021; Jones et al., 2022; Libbrecht, 2017). These effects are predominantly facilitated through the secretion of neurotrophic factors, including brain-derived neurotrophic factor (BDNF) and glial cell line-derived neurotrophic factor (GDNF), which are instrumental in neuronal survival, differentiation, and synaptic plasticity (Park et al., 2020). Furthermore, NSCs exert influence over the neuroimmune milieu by regulating the activity of astrocytes and microglia, thereby modulating both the trajectory of disease progression and the mechanisms of recovery (Matsumoto et al., 2021). Such multimodal therapeutic actions render NSC-based interventions particularly auspicious for addressing acute neural injuries, such as stroke and hypoxic-ischemic encephalopathy, in addition to chronic neurodegenerative conditions.

Concurrently with these mechanistic revelations, substantial advancements have been achieved in the development of innovative sources and derivation methodologies for NSCs. Whereas initial strategies concentrated on the direct isolation from neural tissues, contemporary approaches have embraced differentiation from pluripotent stem cells (PSCs), including induced pluripotent stem cells (iPSCs), as well as the direct transdifferentiation of somatic cells. These methodologies not only mitigate ethical concerns associated with the utilization of embryonic stem cells but also promote the establishment of patient-specific therapies characterized by diminished immunogenicity (Takahashi et al., 2021). Recent investigations have even illustrated the successful isolation of NSCs from cerebrospinal fluid (CSF), thereby offering a minimally invasive alternative for the procurement of stem cells in clinical applications (Friedrich et al., 2020). Moreover, advancements in reprogramming techniques, contingent upon the preservation of genomic and phenotypic stability, present additional prospects for the scalable and ethically sound production of NSCs (Vasileiou et al., 2022). In light of these capabilities, NSC therapies are currently undergoing rigorous investigation for a diverse array of neurological and oncological disorders. In the context of stroke rehabilitation, for example, NSCs have exhibited the potential to repair compromised neural circuits and reinstate lost functionalities (Xu et al., 2020). In the field of oncology, their innate tumor-tropic characteristics have been harnessed to formulate NSC-based delivery systems adept at transporting cytotoxic agents directly to both primary and metastatic brain tumors, thereby augmenting therapeutic efficacy while minimizing systemic adverse effects (Yang et al., 2021; Chen et al., 2022). These dual capabilities position NSCs as both direct therapeutic agents and precision delivery vehicles for supplementary treatments.

Simultaneously, advances in technological innovations about gene editing and cellular engineering have significantly augmented the therapeutic capabilities of neural stem cells (NSCs). Instruments such as CRISPR-Cas9 facilitate the meticulous alteration of NSCs to express designated therapeutic genes, thereby enhancing their neuroprotective and immunomodulatory properties (Zhang et al., 2022a). Furthermore, scholarly attention has increasingly focused on the NSC secretome, which encompasses a complex array of bioactive factors that mediate intercellular signaling and promote tissue repair. Emerging evidence indicates that these secreted factors may be utilized as standalone or adjunct therapeutic interventions (Chung et al., 2021). In conjunction with these developments, advanced imaging modalities, including magnetic resonance imaging (MRI) and *in vivo* fluorescence tracking, provide the capability for real-time observation of NSC migration and integration within the cerebral context. These methodologies are indispensable for assessing the efficacy and safety of NSC-based therapies across both preclinical and clinical frameworks (Lee et al., 2023). Overall, the confluence of mechanistic, technological, and clinical advancements has established NSC therapy at the vanguard of next-generation interventions for neurological and oncological ailments. As our comprehension of NSC biology continues to expand and as translational technologies progress, these therapies are poised to become progressively sophisticated, tailored, and readily available, as illustrated in Table 3.

### Comparative analysis of experimental strategies in neural stem cell therapies

Neural stem cell (NSC) therapies constitute an emergent domain within the therapeutic landscape for neurodegenerative diseases and traumatic brain injuries. These intervention strategies incorporate a heterogeneous spectrum of experimental methodologies, characterized by the origin of NSCs, the techniques employed for their administration, and the modalities utilized to enhance their therapeutic effectiveness. This section presents a comparative examination of these varied methodologies, systematically assessing their respective advantages and inherent drawbacks. The choice of the NSC source serves as a pivotal factor influencing the clinical translatability, safety profile, and therapeutic viability of NSC-centered interventions. The principal sources of NSCs currently under investigation encompass embryonic stem cells (ESCs), induced pluripotent stem cells (iPSCs), and adult-derived NSCs, each offering a distinct array of benefits and limitations. ESCs, noted for their pluripotent nature, possess the capability for extensive self-renewal and differentiation into a multitude of neural cell types, thereby presenting a considerable reservoir for the generation of substantial NSC populations. Nonetheless, the derivation of ESCs necessitates the termination of human embryos, which engenders profound ethical concerns that hinder their widespread acceptance and application across numerous jurisdictions. Additionally, ESC-derived NSCs demonstrate an increased likelihood of immune rejection and tumorigenesis, thereby constraining their immediate clinical applicability (Takahashi et al., 2022; Liu et al., 2018).

Conversely, iPSCs mitigate the ethical challenges associated with ESCs, as they are produced through the reprogramming of adult somatic cells. The potential for autologous derivation of iPSCs confers the significant advantage of reducing the risk of immune rejection. Despite these merits, iPSCs are not devoid of limitations, including a vulnerability to genetic instability and the possibility of tumorigenicity, particularly if the reprogramming process is not fully realized. These inherent risks necessitate comprehensive safety assessments to ascertain their long-term viability in clinical environments (Takahashi et al., 2022). Nonetheless, iPSCs present a promising pathway for the advancement of patient-specific NSC therapies. Adult-derived NSCs, procured directly from neural tissue, offer a more ethically acceptable alternative, as their acquisition does not involve the destruction of embryos. These cells also exhibit a comparatively diminished risk of tumorigenesis relative to their ESC and iPSC counterparts. However, a notable limitation resides in their restricted availability within the adult brain and their diminished self-renewal capacity, which poses significant challenges for the large-scale production requisite for extensive clinical application (Liu B. et al., 2022). Furthermore, adult-derived NSCs generally display a more constrained differentiation potential compared to those derived from pluripotent stem cells.

The emergence of gene editing technologies, particularly CRISPR-Cas9, has facilitated the development of advanced methodologies for the alteration of the characteristics of neural stem cells (NSCs). These innovative tools permit the creation of genetically modified NSCs specifically designed to produce therapeutic biomolecules or to display improved survival and integration proficiency within the host central nervous system. For example, genetic alterations can enhance NSC functionality by promoting the release of neurotrophic factors such as brain-derived neurotrophic factor (BDNF) and glial cell line-derived neurotrophic factor (GDNF), which are vital for neuronal sustenance in neurodegenerative diseases (Takahashi et al., 2022). Notwithstanding its substantial promise, gene editing is accompanied by inherent risks, including the potential for off-target effects, wherein unintended genomic alterations may result in harmful mutations or adverse consequences. These safety apprehensions, particularly concerning the risk of oncogenesis (Zhang et al., 2023), highlight the imperative for thorough preclinical and clinical assessments to determine the safety and efficacy of therapies involving gene-edited NSCs.

The effectiveness of NSC therapies is also fundamentally contingent upon the delivery modality utilized to administer them to the central nervous system. Present methodologies can be broadly categorized into invasive and non-invasive techniques. Invasive delivery approaches typically entail the direct stereotactic injection of NSCs into designated areas of the brain. This technique enables precise localization and has demonstrated efficacy in preclinical animal models of neurological disorders, such as stroke and neurodegenerative diseases. Nevertheless, invasive procedures carry associated risks, including infection, hemorrhage, and the potential for damage to adjacent neural tissue, particularly in vulnerable patient cohorts (Martinez et al., 2021). The necessity for specialized surgical skill and protracted recovery periods further constrains the widespread clinical implementation of these procedures.

In contrast, non-invasive delivery techniques, including intravenous injection, intranasal administration, and cerebrospinal fluid (CSF) infusion, present a less invasive alternative. These strategies alleviate the risks associated with surgical interventions and diminish the reliance on specialized surgical procedures. However, a considerable obstacle to the efficacy of non-invasive delivery methods is posed by the blood-brain barrier (BBB), which can obstruct the effective transit of NSCs to the intended brain regions. Although intranasal and CSF delivery can partially bypass the BBB, these methods frequently yield inferior cell survival and integration rates relative to direct injection (Teng et al., 2021). As a result, non-invasive delivery techniques have generally exhibited less robust therapeutic outcomes in both preclinical and clinical contexts.

While preclinical investigations have indicated substantial potential for NSC-based therapies, the translation of these results into effective human interventions has proven to be a multifaceted challenge. In animal models, NSC transplantation has demonstrated promising outcomes, including enhancements in motor function in Parkinson’s disease models and cognitive improvements in Alzheimer’s disease models, thereby sparking further clinical exploration (Lai et al., 2020). However, human clinical trials have often yielded more modest results, commonly attributed to variables such as limited sample sizes, brief follow-up periods, and difficulties in accurately monitoring NSC survival and integration (Liu H. et al., 2022; Korczyn et al., 2011). Several factors contribute to these mixed clinical results, including immune rejection, suboptimal cell survival within the host environment, and difficulties in achieving appropriate integration into existing neural circuits. Furthermore, the potential for tumor formation, particularly with pluripotent stem cell-derived NSCs, necessitates stringent monitoring and safety protocols in clinical trials (Zhang et al., 2023). The long-term efficacy and safety of NSC therapies in humans remain to be fully established, underscoring the critical need for continued rigorous research and clinical testing. By and large, the many experimental strategies used in NSC-based treatments offer a complicated interaction of benefits and drawbacks. Although ESCs and iPSCs have a great deal of potential for differentiation, safety issues, particularly the possibility of tumorigenicity, and ethical reasons limit their clinical use. Although adult-derived NSCs present a potentially safer and more morally acceptable option in terms of tumor growth, their availability and differentiation capabilities are constrained. Although gene editing has great potential to improve NSC function, long-term safety and off-target effects are still important factors to take into account. Both invasive and non-invasive delivery techniques include trade-offs between accuracy and related dangers. The successful application of NSC therapies to human patients is still a complex task that requires more thorough research into their safety, effectiveness, and long-term effects, despite the encouraging results of preclinical trials, as shown in Table 4.

**TABLE 4:** Summary of Neural Stem Cell Clinical Trials in Neurodegenerative Diseases: A thorough overview of the clinical trials looking into the application of neural stem cells (NSCs) in the management of different neurodegenerative illnesses is provided in this table. This table highlights the potential therapeutic function of NSCs in illnesses including Alzheimer’s disease, Parkinson’s disease, and other age-related neurological disorders by providing comprehensive information on the goals, methods, results, and outcomes of these trials. It provides an overview of the developments in this new subject, highlighting the several tactics and methods being investigated to use NSCs’ capacity for regeneration to enhance neuronal function and impede the course of disease.

Clinical trial focus	Intervention	Findings	References
Neural stem cell therapy for Alzheimer’s disease	Intracerebral transplantation of neural stem cells	Some patients exhibited improvements in cognitive function	Kang et al., 2016; Lai et al., 2020
Neural stem cell therapy for Huntington’s disease	Intracerebral transplantation of neural stem cells	No serious adverse events; patients demonstrated motor function improvements	Gholamzad et al. (2023)
Neural stem cell therapy for Multiple Sclerosis	Intracerebral transplantation of neural stem cells	No serious adverse events; some patients exhibited neurological function improvement	Michailidou et al. (2015)
Safety and efficacy of neural stem cell transplantation in Alzheimer’s disease	Intracerebral transplantation of neural stem cells	No serious adverse events; some patients showed improvements in cognitive function	Park et al. (2020)
Neural stem cell transplantation for Parkinson’s disease	Intracerebral transplantation of neural stem cells	Patients demonstrated improvements in motor function	Madrazo et al. (2019)
Neural stem cell therapy for Parkinson’s disease	Intracerebral transplantation of neural stem cells	No safety concerns; some patients exhibited motor function improvements	Mandel and Korczyn (2012)
Adult neural stem cell therapy for spinal cord injury	Intraspinal transplantation of neural stem cells	Neurological improvement observed with no serious adverse effects	Fan et al. (2017)
Neural stem cell therapy for Amyotrophic Lateral Sclerosis (ALS)	Intraspinal transplantation of neural stem cells	Some patients demonstrated improvements in neurological function	Liu et al. (2022a)

### Limitations and current challenges of neural stem cell-based therapy

Neural stem cell (NSC)-based therapeutic interventions exhibit considerable potential for the treatment of various neurological disorders and injuries. Nevertheless, the effective implementation of such therapies in widespread clinical settings is presently obstructed by numerous formidable challenges (Gao et al., 2020). These impediments include immunological rejection, ethical issues, contamination risks, the need to ascertain treatment efficacy, possible adverse effects, constraints in NSC labeling methodologies, procedural difficulties, intricacies associated with cell purification processes, potential toxicity, and the risk of tumorigenesis. It is imperative to ensure the safety and efficacy of NSC-based therapies. This requires addressing the possibility of tumor development and immune rejection, in conjunction with the establishment of standardized protocols for the isolation, expansion, and differentiation of stem cells. Moreover, the optimization of transplantation parameters—such as the timing of intervention, selection of specific cell types, and route of administration—is critical for the successful clinical translation of these therapies (Zeng C. W., 2023).

Immunological Rejection and Ethical Considerations: Ethical dilemmas and the potential for immunological rejection represent considerable obstacles to the clinical implementation of neural stem cell therapy. The utilization of embryonic stem cells, in particular, elicits substantial ethical concerns owing to the necessity of the destruction of human embryos. Furthermore, obtaining informed consent is vital, necessitating that patients are thoroughly informed of the potential risks and benefits associated with stem cell therapies (Wang et al., 2007; Ying C. et al., 2023). The origin of NSCs significantly influences immunological responses, as allogeneic (donor-derived) cells may provoke rejection in the recipient. Although autologous (patient-derived) NSCs diminish this risk, their preparation may be labor-intensive and financially burdensome, as previously noted.

Risk of Tumorigenicity and Regulatory Challenges: The intrinsic risk of tumorigenicity, combined with the pluripotent differentiation capabilities of NSCs, significantly contributes to the regulatory challenges linked with stem cell therapies (Zhang et al., 2022b). Thorough preclinical and clinical evaluations are indispensable for securing regulatory approval, ensuring meticulous compliance with both ethical standards and evolving regulatory frameworks. The absence of standardized protocols for NSC isolation, characterization, and transplantation further complicates the regulatory milieu, as previously indicated. The establishment of robust and reproducible methodologies is essential for demonstrating the requisite safety and efficacy mandated by regulatory authorities.

Limitations of Current Studies on Neural Stem Cells for Therapeutic Application: Finally, even when NSCs are successfully transplanted into the cerebral environment, their therapeutic efficacy is frequently constrained, and potential adverse effects warrant careful consideration (Gao et al., 2020). Post-transplantation, NSCs may fail to differentiate into the requisite neuronal types, or they may not effectively integrate into existing neural networks, particularly if the inflammatory milieu of the brain undermines their survival and integration (Teng et al., 2021). Investigations have also indicated that NSCs do not invariably facilitate long-term functional recovery. Additionally, challenges such as the blood-brain barrier and the immune environment of the brain can further impede the success of NSC therapies. Furthermore, the methodologies employed to label and monitor transplanted NSCs inherently possess limitations, thereby affecting our capacity to fully comprehend their behavior and integration (Gao et al., 2020). Procedural complications during transplantation and the need for effective cell purification techniques also contribute to the overall challenges. Thus, the full therapeutic potential of NSCs is yet to be realized, and improvements in delivery methods and adjunct therapies are needed to enhance their efficacy while minimizing potential toxicity and other side effects (Gao et al., 2020; Lai et al., 2020; Zeng Y., 2023).

Another pivotal challenge pertains to the safety of neural stem cell (NSC) therapies, particularly concerning their propensity for tumorigenicity and their pluripotent differentiation capacity (Zhang et al., 2022a; Gao et al., 2020). The potential for tumorigenesis remains a major concern, particularly with NSCs obtained from induced pluripotent stem cells (iPSCs), as these cellular entities may retain pluripotency or undergo malignant transformation after transplantation (Zhang et al., 2021; Van Laar et al., 2014; Zeng C. W., 2023). This phenomenon could precipitate the development of teratomas or other neoplasms, thereby posing substantial risks to patients. To alleviate this risk, it is imperative to implement stringent screening protocols and adopt safer differentiation methodologies. Moreover, continuous monitoring of individuals receiving NSC transplants is vital to identify early indicators of tumorigenicity or aberrant cellular proliferation. Ensuring the safety of NSC therapies is of utmost importance for their successful clinical translation, and this aspect constitutes a central focus of regulatory oversight, necessitating comprehensive preclinical and clinical evaluations (Zhang et al., 2022b), as depicted in Table 4.

Immune rejection also constitutes a considerable obstacle in the clinical deployment of NSC therapies, particularly concerning allogeneic (donor-derived) NSCs, and represents a significant ethical consideration (Wang et al., 2007; Ying Z. et al., 2023; Zhang et al., 2022a). Despite the categorization of NSCs as immune-privileged cells, they can still elicit immune responses in the host, particularly if sourced from an external donor (Jones et al., 2023). This response may result in rejection or inflammation at the transplantation site, thereby undermining the therapeutic efficacy. Autologous NSC therapies, which utilize the patient’s cells, may diminish the likelihood of immune rejection. Nonetheless, these therapies are accompanied by their own set of challenges, including the necessity for bespoke cell production for each patient, which is inherently time-consuming and financially burdensome. Innovations in immune modulation strategies, such as the application of immunosuppressive agents or genetic alterations, may assist in addressing these challenges; however, additional research is warranted to refine these methodologies. Furthermore, the risk of contamination during the various phases of cell processing represents a significant safety concern that must be rigorously managed (Gao et al., 2020; Zhang et al., 2022b).

Existing Controversies in the Field: A primary controversy centers around the ethical dilemmas associated with stem cell utilization, especially concerning iPSCs and embryonic stem cells (Wang et al., 2007; Ying C. et al., 2023). While iPSCs provide the benefit of being autologous, thus minimizing the risk of immune rejection, their derivation from embryonic stem cells raises ethical apprehensions regarding the creation and potential destruction of human embryos. Additionally, the capacity for genetic modifications, particularly via gene-editing technologies such as CRISPR-Cas9, has ignited discussions regarding the ethical boundaries of genetic manipulation and the risks of inadvertent genetic alterations (Zhang et al., 2023). These ethical dilemmas, coupled with the potential for modifying human neural tissue, necessitate meticulous consideration and transparent research protocols to navigate the intricate moral landscape, ensuring that informed consent is secured from all participants (Wang et al., 2007; Ying Z. et al., 2023). Another area of controversy lies in the regulatory challenges surrounding NSC therapies due in part to the inherent risks, such as tumorigenicity and the need for robust safety data (Zhang et al., 2022a; Gao et al., 2020). Regulatory bodies, such as the U.S. Food and Drug Administration (FDA) and the European Medicines Agency (EMA), have stringent guidelines for stem cell-based therapies. However, the lack of standardized protocols for NSC isolation, characterization, and transplantation presents difficulties in obtaining regulatory approval. Additionally, uncertainties regarding how these therapies should be classified—whether as drugs, biological products, or medical devices—complicate the regulatory process, leading to delays in approval (Takahashi et al., 2022). These regulatory hurdles can slow the development of NSC therapies and increase costs, limiting their potential for widespread clinical application.

### Perspectives and future directions in neural stem cell therapy

Neural stem cell (NSC) therapy has emerged as a highly promising modality for the treatment of neurodegenerative disorders and cerebral injuries, exhibiting potential efficacy in both preclinical and initial clinical investigations. Considerable progress has been achieved in the derivation of NSCs, encompassing techniques such as direct isolation from cerebral tissue, differentiation from pluripotent stem cells (PSCs), including induced pluripotent stem cells (iPSCs), and transdifferentiation from somatic cells (Tang et al., 2017). Moreover, advancements in gene editing methodologies, notably CRISPR-Cas9, have enabled more refined modifications of NSCs, thereby enhancing their therapeutic potential through improved neuroprotection and immunomodulatory effects (Benmelouka et al., 2021). The advent of neuromodulation-based strategies also presents significant promise in directing NSC differentiation, migration, and integration into host neural circuits, consequently facilitating functional recovery (Yuan et al., 2021). Non-invasive imaging modalities have further propelled the field forward by providing enhanced capabilities for the monitoring of NSC fate and integration *in vivo*, thus yielding valuable insights into therapeutic outcomes (Reekmans et al., 2011). These advancements lay a critical foundation for ongoing clinical trials designed to evaluate the safety and efficacy of NSC-based interventions for a variety of neurological disorders.

Notwithstanding these advancements, a multitude of challenges persist that impede the broader clinical implementation of NSC therapies. A primary concern pertains to the variability evident in preclinical findings, which complicates the translatability of these therapies into clinical applications. The characteristics of NSCs, encompassing their source, engineering methodologies, and delivery techniques, significantly influence therapeutic success, frequently resulting in heterogeneous outcomes across diverse studies (Reekmans et al., 2011). Furthermore, the fundamental mechanisms through which NSCs facilitate recovery, particularly in intricate conditions such as ischemic stroke and neurodegenerative diseases, remain inadequately understood, thereby constraining efforts to optimize therapeutic strategies (Mattson, 2001; Zhao et al., 2022). An additional challenge involves ensuring the long-term survival, integration, and functional contribution of transplanted NSCs within the recipient brain (Zeng Y., 2023). Safety concerns, including tumorigenicity, immunogenicity, and potential adverse effects stemming from genetic modifications, also represent critical issues that necessitate resolution (Deokate et al., 2024a). Furthermore, although non-invasive delivery techniques such as intranasal and cerebrospinal fluid (CSF) injections proffer advantages relative to patient safety, they continue to encounter substantial barriers, including the necessity to traverse the blood-brain barrier (BBB) and guarantee targeted delivery to the injury site. Invasive transplantation techniques, while offering greater precision, introduce risks associated with surgical complications, such as infection and damage to adjacent healthy brain tissue. Ethical dilemmas regarding the utilization of embryonic stem cells (ESCs) and genetic modifications to NSCs further complicate the clinical translation of these therapies, necessitating meticulous consideration of potential unintended consequences (De Gioia et al., 2020).

Subsequent investigations must prioritize the resolution of these challenges to optimize neural stem cell (NSC) therapies. A fundamental aim is to establish standardized protocols for the isolation, characterization, and transplantation of NSCs, which will facilitate the reduction of variability and guarantee consistent outcomes across clinical trials (Tang et al., 2017). The incorporation of advanced tissue engineering methodologies and biomaterials has the potential to enhance the viability and integration of transplanted NSCs within the host cerebral environment, thereby fostering a more favorable milieu for their differentiation and functional rehabilitation (Yuan et al., 2021). Tailored approaches, such as the utilization of patient-specific induced pluripotent stem cells (iPSCs) derived from individual somatic cells, exhibit considerable promise in diminishing immune rejection and customizing therapies by distinct genetic profiles, thereby ensuring superior therapeutic efficacy (Zhao et al., 2022). Furthermore, comprehensive long-term safety assessments are imperative to evaluate potential hazards, including tumorigenicity and immune responses, along with any unforeseen adverse effects associated with NSC therapies (Deokate et al., 2024b).

Emerging technologies, encompassing gene editing and biomaterials, present auspicious pathways for augmenting the therapeutic efficacy of NSCs while alleviating safety apprehensions (Benmelouka et al., 2021; Zeng C. W., 2023). For instance, gene editing instruments such as CRISPR-Cas9 may be employed to meticulously alter NSCs to express therapeutic proteins or to enhance their capacity to assimilate into host neural circuits. Additionally, innovations in biomaterials could engender a supportive microenvironment for NSCs, thereby promoting their survival and differentiation post-transplantation. Collaborative initiatives spanning various disciplines—including research, clinical practice, bioengineering, and industry—are crucial for expediting the transition of NSC therapies from laboratory settings to clinical applications (De Gioia et al., 2020; Ying C. et al., 2023). Persisting in the exploration of the NSC secretome—the collection of bioactive factors secreted by NSCs that modulate tissue repair—may also yield significant insights into the role of these cells in healing and improving therapeutic outcomes (Benmelouka et al., 2021).

In conclusion, although NSC therapy presents substantial promise for the treatment of neurological disorders, surmounting the challenges related to safety, delivery, and clinical variability is vital for its broader clinical implementation. Continuous research aimed at refining NSC derivation techniques, bolstering cell survival and integration, and addressing safety issues will be pivotal in actualizing the complete therapeutic potential of NSC-based interventions. With sustained innovation and interdisciplinary cooperation, NSC therapies may ultimately offer effective, safe, and accessible treatment options for a variety of neurological conditions.

## Abbreviations

SVZ, subventricular zone; GFAP, Glial fibrillary acidic protein; RMS, rostral migratory stream; OB, olfactory bulb; SGZ, subgranular zone; DG, dentate gyrus; BLBP, brain lipid binding protein; VEGF, vascular endothelial growth factor; ALS, amyotrophic lateral sclerosis; GDNF: glial cell line-derived neurotrophic factor, ESCs: embryonic stem cells; iPSCs, induced pluripotent stem cells; NSCs, neural stem cells; FDA, Food and Drug Administration; EMA, European Medicines Agency.
